# Ca^2+^ signaling and metabolic stress-induced pancreatic β-cell failure

**DOI:** 10.3389/fendo.2024.1412411

**Published:** 2024-07-02

**Authors:** Mark A. Magnuson, Anna B. Osipovich

**Affiliations:** Department of Molecular Physiology and Biophysics and Center for Stem Cell Biology, Vanderbilt University, Nashville, TN, United States

**Keywords:** type 2 diabetes, pancreatic β-cells, Ca^2+^ signaling, metabolic stress, dedifferentiation, gene expression

## Abstract

Early in the development of Type 2 diabetes (T2D), metabolic stress brought on by insulin resistance and nutrient overload causes β-cell hyperstimulation. Herein we summarize recent studies that have explored the premise that an increase in the intracellular Ca^2+^ concentration ([Ca^2+^]_i_), brought on by persistent metabolic stimulation of β-cells, causes β-cell dysfunction and failure by adversely affecting β-cell function, structure, and identity. This mini-review builds on several recent reviews that also describe how excess [Ca^2+^]_i_ impairs β-cell function.

## 1 Introduction

T2D is a polygenic disease in which insulin resistance brought on by overnutrition, obesity, age, and a high genetic risk profile leads to the loss of glycemic control (1, 2). While many individuals exhibit insulin resistance, the loss of β-cell function in response to mounting metabolic stress determines whether euglycemia is maintained. Thus, to understand the pathogenesis of T2D, we must also know how the β-cell responds to metabolic stress and why it fails, especially in the pre-diabetic stage of the disease.

Compelling evidence exists that endoplasmic reticulum (ER) stress (3), mitochondrial dysfunction (4), cytokine signaling (5) and the loss of cell identity (6) all contribute to the loss of β-cell function in response to metabolic stress. Moreover, over 600 genetic risk loci for T2D have been identified by genome-wide association studies (GWAS) (7–9). While the target genes for most risk loci have not been unambiguously determined, islet-specific transcription factors often bind nearby, suggesting in many cases that they predispose β-cells to fail (10–12).

To develop a mechanism-based explanation for β-cell failure that integrates both genetic and biochemical knowledge, we build on several recent reviews (13–17) and summarize recent studies that point to the dysregulation of intracellular Ca^2+^ concentrations ([Ca^2+^]_i_) as a unifying explanation for the seemingly diverse mechanisms and genes that may contribute to β-cell failure in response to metabolic stress.

## 2 Metabolic stimuli-insulin secretion coupling

**Figure 1:**
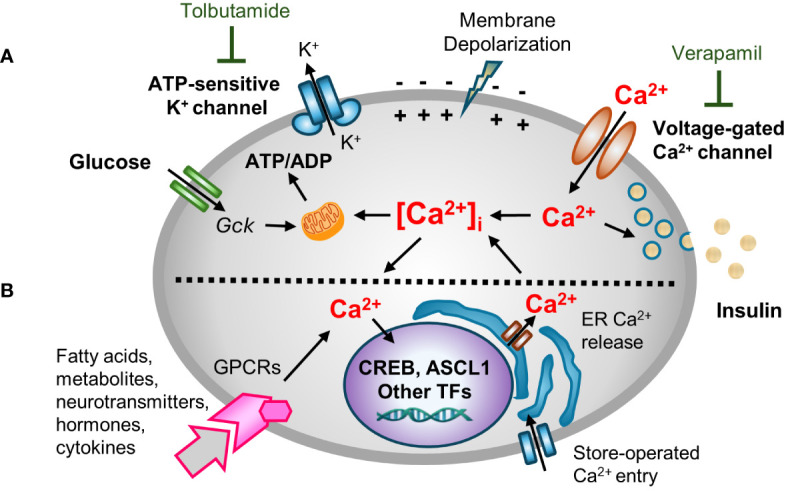
A multifaceted role for [Ca^2+^]_i_ in β-cell function. Hyperglycemia and other insulin secretagogues cause [Ca^2+^]_i_ in β-cells to increase. **(A)** Metabolism-stimulated insulin secretion. Glucose and amino acid metabolism lead to an increase in the ATP/ADP ratio, causing K_ATP_ (ATP-sensitive) potassium channel closure, plasma membrane depolarization, and the opening of voltage‐gated Ca^2+^ channels (VDCC) triggering transient increases in intracellular Ca^2+^ concentrations ([Ca^2+^]_i_). The transient spikes in [Ca^2+^]_i_ stimulate docking and exocytosis of insulin vesicles. The activity of K_ATP_ and VDCC channels can be blocked with tolbutamide (a sulfonylurea) and verapamil, respectively. **(B)** Adaptive regulation. Hormones, cytokines, neurotransmitters, and certain fatty acids and metabolites that signal through G protein-coupled receptors (GPCRs) activate intracellular signal transduction pathways that cause Ca^2+^ efflux/influx from intracellular stores/extracellular space. GPCR-induced alterations in [Ca^2+^]_i_ modify other metabolism-based insulin secretory responses. In addition, Ca^2+^ signaling to the nucleus alters the expression Ca^2+^-dependent transcription factors that control many cellular functions.

Ca^2+^ is a critical second messenger that regulates many cellular processes in β-cells, insulin exocytosis being foremost among them (18). Decades of studies have provided a now canonical model for metabolism-stimulated insulin secretion (19). Briefly, and as illustrated in **Figure 1A**, a rise in the plasma glucose concentration is sensed by the β-cell through the metabolism of glucose leading to an increase in the cellular ATP/ADP ratio. The rise in ATP/ADP ratio causes ATP-sensitive potassium (K_ATP_) channel closure, plasma membrane depolarization, the opening of voltage‐gated Ca^2+^ channels (VDCC), and a rise in intracellular Ca^2+^ concentrations ([Ca^2+^]_i_). The transient spikes in [Ca^2+^]_i_ stimulate docking and exocytosis of insulin vesicles (20).

Blocking K_ATP_ channel activity, either by the administration of sulfonylureas or by genetic disruptions, causes depolarization of the plasma membrane. Conversely, use of diazoxide, a molecule that opens the K_ATP_ channel, or expression of K_ATP_ channel subunits (encoded by *Kcnj11* and *Abcc8*) that contain activating mutations, causes hyperpolarization of the plasma membrane (21–24). However, while the canonical model nicely links cell metabolism to insulin secretion and predicts an increase in [Ca^2+^]_i_ in response to metabolic stress, it overlooks the now well-established fact that [Ca^2+^]_i_ affects other β-cell organelles, such as the ER, mitochondria, and nucleus (15, 25, 26). Moreover, while the rise of [Ca^2+^]_i_ is principally metabolism-driven, insulin exocytosis is modulated by many other agents including many hormones, fatty and amino acids, and neurotransmitters in so-called “amplifying pathways”. Many of these agents act by binding to G protein-coupled receptors (GPCRs), and the activation of phospholipase C (PLC)/protein kinase C (PKC) or adenylate cyclase (AC)/protein kinase A (PKA) pathways which rely on Ca^2+^ for signaling (27–30). An extended model (**Figure 1B**) recognizes both the role of other agents in modulating insulin secretion, and the critically important role of Ca^2+^ in other organelles.

### 2.1 Ca^2+^ is essential for many functions of the β-cell

[Ca^2+^]_i_ is tightly regulated by transmembrane channels and pumps, Ca^2+^ buffering proteins, and by the uptake and release of Ca^2+^ from ER stores and mitochondria (31). Ca^2+^ concentrations vary considerably among different subcellular compartments, with concentrations in extracellular space (~1–2 mM) and ER and Golgi compartments (~200–700 µM) being more than ten thousand times higher than in the cytosol (~100 nM) (32, 33). Mitochondria Ca^2+^ concentrations vary between 50–500 nM in order to regulate metabolism and serve as a transient calcium buffer (34). Metabolic stimulation causes [Ca^2+^]_i_ to sharply increase from basal levels of ~100 nM to stimulated levels of ~1–3 µM due to Ca^2+^ entry from the extracellular space or Ca^2+^ release from intracellular stores. These spikes in [Ca^2+^]_i_ may start locally before propagating as cyclical Ca^2+^ oscillations throughout the islet (35, 36). While Ca^2+^ spikes are tightly linked to insulin secretion, an increase in [Ca^2+^]_i_ also directly affects Ca^2+^ concentrations in various organelles, including the nucleus, through multiple influx/efflux pathways (37).

### 2.2 Role of Ca^2+^ in the ER and mitochondria

Both the ER and mitochondria require Ca^2+^ for their function, and both serve as intracellular Ca^2+^ reservoirs. The ER is critical for protein synthesis and folding, lipid synthesis, and Ca^2+^ storage and release, and β-cells require optimal ER functionality to support the production of insulin and to maintain [Ca^2+^]_i_ homeostasis (25). The entry and release of Ca^2+^ from and to the ER is mainly regulated by SERCA pumps, or by inositol 1,4,5-triphosphate (IP3R) and by ryanodine receptors (RyR), respectively. Moreover, store-operated Ca^2+^ entry from the extracellular space plays a critical role in maintaining ER Ca^2+^ concentrations. Dysfunctions in any of these processes can change ER susceptibility to stress (38, 39). Not only does ER Ca^2+^ affect the unfolded protein stress response (40), persistent ER stress likely causes β-cell demise in both Type 1 and T2D (25, 41). Indeed, cytokine-induced depletion of Ca^2+^ from the ER may directly trigger apoptosis (42).

Mitochondrial activity and metabolic enzymes are also regulated by Ca^2+^ (43, 44). Glucose-stimulated insulin secretion is directly linked to mitochondrial function, and Ca^2+^ flows between the ER and mitochondria via mitochondria-associated ER membrane (MAMs) contact sites. Since Ca^2+^ is released from the ER and directly taken up by mitochondria, intraluminal ER, mitochondrial matrix, and cytoplasmic Ca^2+^ concentrations are closely interrelated (45, 46). ER-mitochondria interplay may also be a critical cellular adaptive mechanism for restoring [Ca^2+^]_i_ homeostasis after episodes of Ca^2+^ overload, thereby also contributing to β-cell dysfunction (47, 48).

### 2.3 Ca^2+^ signaling to the nucleus

Ca^2+^ signaling to the nucleus links signaling cues with gene expression, enabling cellular adaptations to both internal and external stimuli (49). This process, known as excitation-transcription coupling, is well-described in neurons and myocytes (50–53). Ca^2+^ influx in response to membrane depolarization and GPCR activation triggers multiple intracellular signaling pathways that regulate cell identity, proliferation, autophagy and cell death (54). Glucose stimulation leads to increase in β-cell nuclear Ca^2+^ concentration (55, 56). [Ca^2+^]_i_ is sensed by Ca^2+^-binding proteins (CBPs), with calmodulin being the most well-studied (57). These molecules in turn activate downstream targets, including the protein phosphatase calcineurin and Ca^2+^/calmodulin-dependent kinases (e.g. CamKII and CamKIV*)*, which in turn modulate the activity of Ca^2+^ responsive transcription factors such as NFAT and CREB (58–62), as well as a number of other transcription factors, transcriptional co-regulators and chromatin modifying enzymes (63, 64). While excitation-transcription coupling is a part of a normal response to changes in the cell environment, chronic and sustained activation of Ca^2+^ signaling pathways is detrimental to cell function (65–67).

### 2.4 Evidence linking Ca^2+^ signaling to β-cell failure

**Figure 2:**
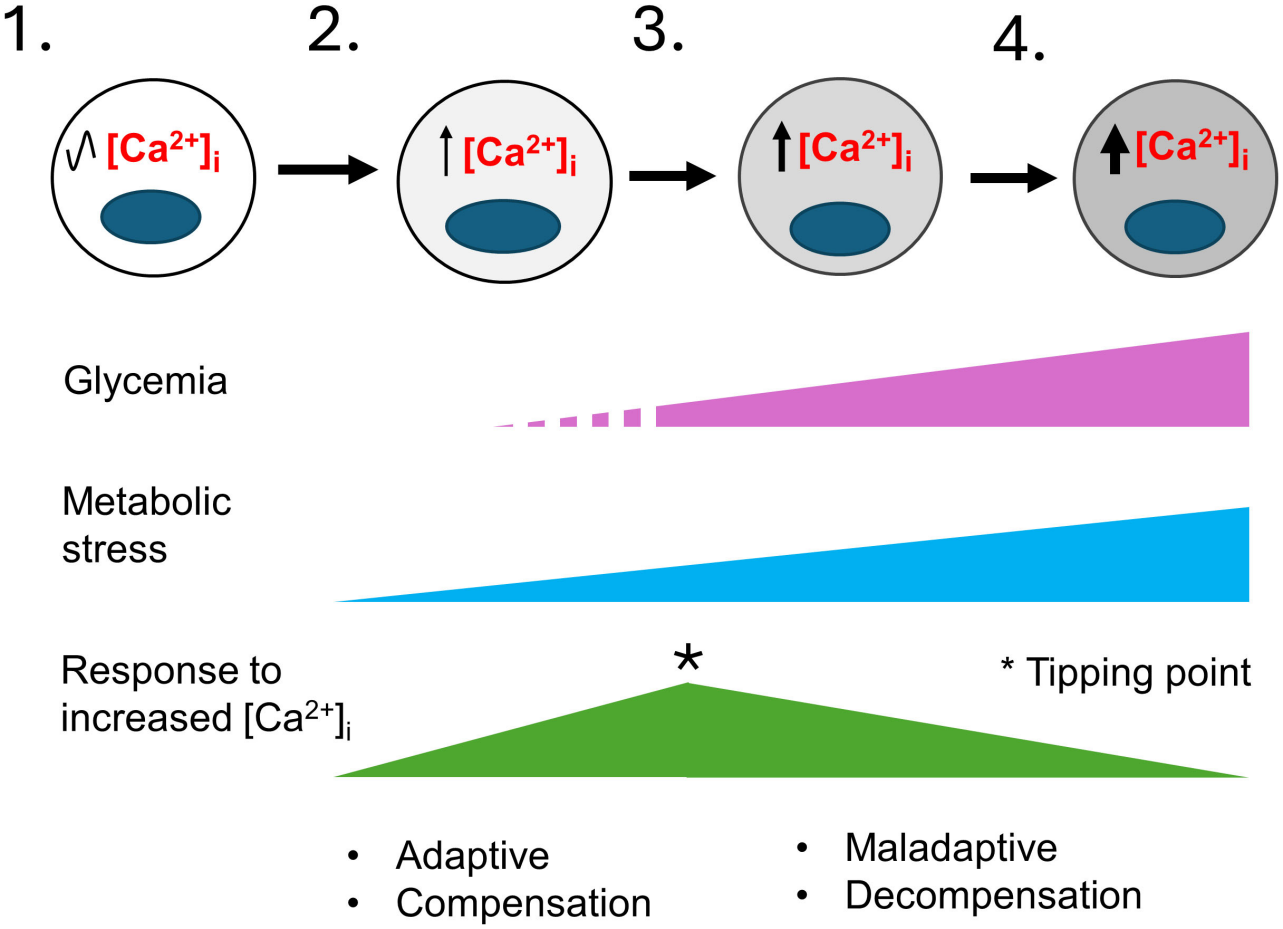
A model for the development of T2D that considers the role of metabolic-stress and [Ca^2+^]_i_. This model illustrates the stepwise failure of β-cells in response to metabolic stress. 1) In healthy individuals β-cells have normal [Ca^2+^]_i_ levels. 2) In pre-diabetes, environmental factors such as age, sex, genetic makeup, obesity, and overnutrition cause insulin resistance and an increase in insulin demand resulting in mild metabolic stress. Transient elevations of the blood glucose and other insulin secretagogues cause the hyperstimulation of β-cells and small increases in [Ca^2+^]_i_. Initially, the increase in Ca^2+^-signaling stimulate insulin secretion, β-cell proliferation and other adaptive responses that continue to maintain glycemia and compensate for increased insulin demand. 3) The limited ability of β-cells to compensate together with a continuing rise in metabolic stress cause further increases in [Ca^2+^]_i_. A tipping point occurs where a network of Ca^2+^-regulated genes crucial for maintaining Ca^2+^ homeostasis and β-cell function becomes maladaptive. 4) The maladaptive changes brought on by chronically elevated [Ca^2+^]_i_ cause the loss of β-cell identity and function, with β-cells entering a decompensation stage where they can no longer secrete enough insulin to maintain normal blood glucose. Glucolipotoxicity further accelerates the loss of β-cell function, identity and viability, therefore resulting in overt T2D. The increased gray shading of β-cells from left to right indicates increasing loss of β-cell identity and function. The * indicates a tipping point.

While the harmful effects of excess [Ca^2+^]_i_ have been extensively investigated in other excitable cell types, and a critical role for Ca^2+^ signaling in β-cell function has long been clear, we lack a clear understanding of how Ca^2+^ signaling is linked to β-cell failure (13–17). Prior studies have shown that basal [Ca^2+^]_i_ is increased in rat islets cultured in high glucose (68), in mouse islets from obese (*db/db*) mice (69–71), in islets from mice fed a high-fat diet (HFD) (72), and in β-cells that exhibit chronic membrane depolarization due to the loss of K_ATP_ channel subunit *Abcc8* (73). Conversely, lowering [Ca^2+^]_i_ in β-cells by blocking Ca^2+^ influx with verapamil (74–78), other compounds (79), or by a genetic deletion of *Cavβ3*, a voltage-dependent calcium channel subunit (80), attenuates β-cell loss and diabetes in mice and humans. Moreover, studies in which β cells are “rested” through the use of diazoxide, which opens the K_ATP_ channel thereby impairing membrane depolarization, or other therapies that lower the blood glucose concentration or reduce glucokinase activity, may all work in large part by limiting increases in [Ca^2+^]_i_ (41, 71). However, while there is ample evidence showing a correlation between a sustained increase in [Ca^2+^]_i_ and impairments in β-cell function, we do not know how metabolic stress-induced increases in [Ca^2+^]_i_, which are likely to only be transient during the pre-diabetic phase of T2D, initiate events that lead to β-cell failure, as illustrated in **Figure 2**. Similarly, we do not know how specific Ca^2+^-regulated processes are affected by T2D-associated risk loci, either individually or in combination.

## 3 Effects of metabolic stress related increases in [Ca^2+^]_i_ on pancreatic β-cell gene expression

### 3.1 Models of excess Ca^2+^ signaling in β-cells

Consistent with the canonical model (**Figure 1**), depolarization of the plasma membrane is predicted to cause Ca^2+^ influx, a rise in [Ca^2+^]_i_, and an increase in insulin secretion. *Abcc8* and *Kcnj11* knockout mice, both of which lack functional K_ATP_ channels and exhibit a sustained elevation in [Ca^2+^]_i_, display mild hypoglycemia as young animals but develop diabetes as they age (73, 81–85). Similarly, in humans, several individuals with inactivating mutations of the K_ATP_ channel that cause hyperinsulinism in infancy have been reported to develop diabetes in adolescence (86, 87). The findings that both mice and humans with genetically-driven increases in [Ca^2+^]_i_ maintain euglycemia for several months before they cross over to being overtly diabetic has important implications (73, 82). First, it clearly separates the effects of a sustained pathological increase in [Ca^2+^]_i_, often referred to as excitotoxity, from glucotoxicity, which occurs after the onset of hyperglycemia. Second, the delay has enabled studies of how β-cell gene expression and function is affected by a chronic increase in [Ca^2+^]_i_ without the confounding effects of hyperglycemia (73, 82, 88).

### 3.2 Overlapping effects of excitotoxicity and overnutrition

Studies of *Abcc8* knockout mice revealed alterations in islet morphology and glucose intolerance prior to the development of hyperglycemia. In addition, they revealed a loss of β-cell identity that correlated with a marked alteration of a network of Ca^2+^ regulated genes (73). Recently, studies of β-cell-specific *Kcnj11* knockout mice, which also exhibit an increase in [Ca^2+^]_i_ and glucose intolerance, revealed a Gs/Gq signaling switch (89).

Since overnutrition is common in pre-diabetes, we compared transcriptomes of FACS-purified β-cells of *Abcc8* knockout mice, which serve as a model for excitotoxicity, with mice fed a HFD (88). Both excitotoxicity and overnutrition were found to affect overlapping sets of genes, and to exert an additively negative effect on β-cell function (88). The commonalities in transcriptional response are not surprising since overnutrition, by elevating circulating free fatty acids (FFAs), contributes to insulin resistance and hyperglycemia (90) by causing the release of Ca^2+^ from ER stores, increase in [Ca^2+^]_i_ and accentuating both ER and oxidative stress (16, 91–95).

While excitotoxicity and overnutrition individually perturb the expression in β-cells of several thousand genes (88), a meta-analysis revealed that many of the upregulated genes were involved in oxidative phosphorylation, mitochondrial organization, metabolic pathways, and oxidative stress response whereas downregulated genes were involved in cell organization, secretory function, cell adhesion, cell junctions, cilia, cytoskeleton, and regulation of β-cell epigenetic and transcriptional program (88). Furthermore, many genes that are dysregulated excitotoxicity and overnutrition are altered in pre-diabetic and diabetic β-cells from db/db mice (96), also suggesting a strong correlation between chronic alterations in [Ca^2+^]_i_ and the loss of β-cell function.

### 3.3 Transcriptomic changes that precede β-cell failure

#### 3.3.1 Mitochondrial function and energy metabolism

A chronic increase in [Ca^2+^]_i_ stimulates expression of mitochondrial structural and metabolic genes (*Me3*, *Cox7a*) that is parallelled by increased oxygen consumption and mitogenesis in islets (88). Chronic stimulation of the electron transport chain leads to an increase in reactive oxygen species and to mitochondrial dysfunction (97). Furthermore, the combination of increased [Ca^2+^]_i_ and overnutrition not only impairs mitochondrial function, but it may also impair the replacement of metabolically damaged mitochondria (88) due to the downregulation of mitophagy associated genes (*Clec16a*, *Prkn*) (88, 98–100).

Lysosomes are of note since they are involved in maintaining the mitochondrial biogenesis/mitophagy balance (101), and stressed β-cells showed an increase in regulators for mitochondrial (*Ppargc1a*) and lysosomal (*Tfeb*) biogenesis (88), both known to be activated by Ca^2+^ in other cell types (102, 103). *Ppargc1a*, a key transcriptional regulator of energy metabolism, FA β-oxidation and mitochondrial biogenesis is implicated in β-cell dysfunction and T2D (104–106).

Metabolic stress, by increasing the expression of genes that contribute to metabolic inflexibility, may also impair the ability of β-cells to utilize glucose, which would impair their metabolic response to glucose (107). Consistent with this, an increase in the expression of FA β-oxidation genes, as well as *Pdk4*, a kinase that inhibits pyruvate flux into the TCA cycle (108), and decrease in mitochondrial respiration response to glucose also suggests that stressed β-cells switch to FAs and ketones as mitochondrial fuels (88).

Together, these findings suggest that metabolic-stress induced elevations in [Ca^2+^]_i_ cause impairments in mitochondrial function that reduce the ability of β-cells to respond to glucose, an unambiguous sign of β-cell failure.

#### 3.3.2 ER protein folding and protein glycosylation

Excitotoxicity and overnutrition also cause an increase in the expression of genes associated with ER secretory stress (88, 109). Glycosylation, the process during which glycans (mono- or oligosaccharides) are attached to proteins in the ER and Golgi, is a critical quality control signal in ER protein folding (110). Since excess protein glycosylation brought on by ER stress is linked to cellular apoptosis (111), the observed increases in expression of genes associated with ER protein folding and N- and O-linked protein glycosylation are highly noteworthy as they suggest that the stability, localization, trafficking, and function of glycosylated receptors, ion channels, nutrient transporters, and transcription factors in β-cells may all be adversely affected (112).

#### 3.3.3 β-cell structure: cytoskeleton, cell polarity, and cell adhesion

Since islet architecture in *Abcc8* knock-out mice is abnormal (73), it is not surprising that many genes important for β-cell structure and function downregulated (88), including those necessary for cell adhesion and cell-cell junctions (113), cilia (114), and cytoskeletal and vesicular trafficking (115). Our transcriptomic analysis also predicts changes in β-cell polarity since genes essential for apical domain formation, primary cilia, and the lateral domain are all downregulated while genes associated with the vasculature-facing basal domain are upregulated (116). Together, the many changes we observed suggest that a chronic increase in [Ca^2+^]_i_ disrupts cell polarity, exocytotic machinery, and critical cell-cell contacts, thereby physically disrupting islet architecture and insulin secretion.

#### 3.3.4 Chromatin maintenance and β-cell identity

Cellular dedifferentiation and the resulting loss of β-cell identity are fundamentally important contributors to β-cell dysfunction in T2D (117, 118). In β-cells that are stressed by excitotoxicity and overnutrition, many transcription factors that are essential for maintaining β-cell identity are downregulated (88). Similarly, epigenetic modifiers, including a DNA methyltransferase (*Dnmt1*) important for silencing of developmental or “disallowed” metabolic genes in mature β-cells (119), are decreased. This likely explains the upregulation of multiple disallowed genes (120) in response to metabolic stress (88). Several lncRNAs which contribute to the maintenance of the epigenetic and transcriptional landscape of β-cells (121), are also down-regulated. Importantly, *Aldh1a3* (122) and *Bach2* (123), two well-established markers and drivers of β-cell dedifferentiation, are upregulated, suggesting that they too are regulated by Ca^2+^ (88). Thus, the continued expression of key transcription factors necessary to maintain β-cell identity likely also depends on the maintenance of Ca^2+^-signaling and homeostatic regulation.

### 3.4 Role of *Ascl1*, a Ca^2+^-regulated gene, in β-cell dedifferentiation and failure

While the expression of many genes in β-cells is altered by metabolic stress, the chronic nature of the analyses performed to date limits the establishment of direct cause-and-effect relationships. For this reason, we sought to identify a Ca^2+^-regulated gene that could be studied in detail. *Ascl1* (*Achaete-scute* homolog 1) stood out since many of the genes putatively upregulated by [Ca^2+^]_i_ contain binding sites for ASCL1 (73). In addition, *Ascl1* is a pioneer transcription factor critical for neural cell differentiation (124–126) and is necessary for the formation of neuroendocrine cells in multiple tissues (127–129). Importantly, *ASCL1* is also expressed in human islets (130).

To investigate how *Ascl1* contributes to β-cell dysfunction during metabolic stress, we generated β-cell-specific *Ascl1* knockout mice and studied their responses to both excitotoxicity and overnutrition. We found that *Ascl1* is indeed induced by stimuli that cause Ca^2+^-signaling to the nucleus, and that it contributes in multiple ways to the loss of β-cell function. Remarkably, the removal of *Ascl1* from β-cells improved their function in response to metabolic stress by HFD feeding (131). Transcriptional profiling of islets under different experimental conditions revealed that ASCL1 contributes to a loss of β-cell function both by activating a dedifferentiation program and by suppressing the expression of secretory and innervation genes in response to overnutrition.

Interestingly, β-specific *Ascl1* knockout islets from HFD mice have increased expression of parasympathetic neuronal markers, increased insulin secretion in response to acetylcholine, and an increased islet innervation. While additional studies of the role of *Ascl1* in stressed β-cells are necessary, our experiments clearly demonstrate that a metabolic stress-induced increase in Ca^2+^-signaling to the nucleus alters both β-cell function and identity in an ASCL1-dependent manner. Our studies also point to a role for other Ca^2+^-regulated transcription factors, suggesting that a Ca^2+^-dependent gene regulatory network is critical for the proper function of β-cells, and that metabolic-stress profoundly modifies this network by invoking both adaptive and maladaptive transcriptional changes.

## 4 Discussion

Although multiple lines of evidence point to Ca^2+^-signaling being intimately involved in metabolic stress-induced β-cell failure, the mechanisms whereby an increase in [Ca^2+^]_i_ leads to a loss of β-cell function are not understood and need further investigation.

1. Temporal causality between changes in [Ca2+]i, the expression of key transcription factors, and the loss of β-cell function needs to be established.
2. We need to better understand how specific Ca^2+^-regulated processes modulate β-cell function. Cleverly designed studies are required to distinguish between the effects of elevated metabolic flux and closely linked [Ca^2+^]_i_.
3. We need to determine how overnutrition and insulin resistance affect Ca^2+^ spiking activity in pre-diabetic setting.
4. We need to better understand how Ca^2+^-mediated transcriptional reprogramming impairs β-cell function and identity.
5. Finally, we need to determine how specific T2D genetic risk loci affect Ca^2+^-dependent processes in β-cells.

While our assertions for the importance of Ca^2+^-signaling have strong experimental support, we do not understand how genetic risk loci, either individually or in aggregate, may contribute to β-cell dysfunction.

We hope that this mini-review stimulates investigations by others as there is much to learn about how alterations in [Ca^2+^]_i_ affect β-cell function and contribute to T2D.

## Author contributions

MM: Writing – original draft, Writing – review & editing. AO: Writing – original draft, Writing – review & editing.
